# Tumorspheres but Not Adherent Cells Derived from Retinoblastoma Tumors Are of Malignant Origin

**DOI:** 10.1371/journal.pone.0063519

**Published:** 2013-06-24

**Authors:** Wesley S. Bond, Patricia Y. Akinfenwa, Laszlo Perlaky, Mary Y. Hurwitz, Richard L. Hurwitz, Patricia Chévez-Barrios

**Affiliations:** 1 Interdepartmental Program in Translational Biology and Molecular Medicine, Baylor College of Medicine, Houston, Texas, United States of America; 2 Department of Pediatrics, Baylor College of Medicine, Houston, Texas, United States of America; 3 Center for Cell and Gene Therapy, Houston, Texas, United States of America; 4 Texas Children's Cancer and Hematology Centers, Houston, Texas, United States of America; 5 Department of Pathology and Genomic Medicine, The Methodist Hospital, Houston, Texas, United States of America; 6 The Retinoblastoma Center of Houston, Houston, Texas, United States of America; Sapporo Medical University, Japan

## Abstract

Verification that cell lines used for cancer research are derived from malignant cells in primary tumors is imperative to avoid invalidation of study results. Retinoblastoma is a childhood ocular tumor that develops from loss of functional retinoblastoma protein (pRb) as a result of genetic or epigenetic changes that affect both alleles of the *RB1* gene. These patients contain unique identifiable genetic signatures specifically present in malignant cells. Primary cultures derived from retinoblastoma tumors can be established as non-adherent tumorspheres when grown in defined media or as attached monolayers when grown in serum-containing media. While the *RB1* genotypes of tumorspheres match those of the primary tumor, adherent cultures have the germline *RB1* genotype. Tumorspheres derived from pRb-negative tumors do not express pRb and express the neuroendocrine tumor markers synaptophysin and microtubule-associated protein 2 (MAP2). Adherent cells are synaptophysin-negative and express pRb, the epithelial cell marker cytokeratin that is expressed in the retinal pigmented epithelium and the vascular endothelial cell marker CD34. While tumorspheres are of malignant origin, our results cast doubt on the assumption that adherent tumor-derived cultures are always valid *in vitro* models of malignant cells and emphasize the need for validation of primary tumor cultures.

## Introduction

Cells derived from primary tumors are commonly used as models for cancer research including for high-throughput genomic and transcriptomic analysis [1] and evaluation of therapeutics for treatment of cancer [2]. In the era of personalized medicine, the use of these primary tumor cells to characterize individual patient tumors will increasingly dictate treatment strategies, as is the case in clinical management of breast cancer [3]. In many instances, primary cultures are not validated genetically and are assumed to be derived from the original malignancy. Examples of years of research being invalidated due to misidentification of cultured cancer cells demonstrate the potential risks and highlight the need for verification of the origin of these cells [4]–[6].

The sphere-forming assay, a culture technique in which aggregates of cells form highly regular spheroid architectures in suspension, is a commonly used *in vitro* method for the study of cultured stem cells [7]–[9] and tumor cells in a variety of malignancies [10]–[13]. The aggregates are thought to be the result of tumor-initiating cells that proliferate and differentiate into the plurality of cell types found in the original tumor [10]. However, the formation of tumorspheres commonly requires specific culturing conditions, such as the use of stem cell-optimized media with defined supplements [14]. In contrast, culturing tumor cells in serum-containing medium can yield cells with markedly different morphologies and growth characteristics. For instance, in a SV40 T-antigen transgenic mouse model of Rb, culturing of tumor cells in medium containing serum typically yields a population of cells with a different phenotype from tumorspheres: an attached monolayer [15]. The true identity of these different primary tumor cultures and definitive knowledge of their origin remain poorly understood.

Retinoblastoma (Rb), the most common intraocular tumor in children, provides an advantageous cancer model with which to study the origin of cells derived from patient tumors. This advantage is due to a specific requisite genetic change in the etiology of the vast majority of Rb tumors: the loss of functioning retinoblastoma protein (pRb) often due to mutation or epigenetic silencing of its coding gene, *RB1* [16]. In hereditary Rb, one allele of *RB1* in the germline contains a loss-of-function mutation. During retinal development, function of the remaining normal allele is lost *de novo* either through mutation, epigenetic silencing or chromosomal nondisjunction, creating a progenitor cell that ultimately produces a retinal tumor. In spontaneous Rb (the more common form), loss of function of both *RB1* alleles occurs *de novo*. Regardless of type, mutations in *RB1* among the patient population are spread widely along the gene, with limited clustering at specific hotspots coinciding with CpG methylation site-related genetic instability [17]. This variability in *RB1* mutations typically leads to a relatively unique mutation in the gene for each Rb patient. The well-defined etiology of Rb oncogenesis and the relative uniqueness of mutations in *RB1* permit straightforward determination of whether cells isolated from a particular Rb patient derive from the germline or from the malignant cell of origin. In this study, we sought to determine whether cultures derived from Rb patient tumors originate from the germline of the patient or from the original malignant cell.

## Materials and Methods

### Antibodies

Antibody against pan-cytokeratin (OSCAR clone) was obtained from Signet Laboratories (Dedham, MA). Antibodies against synaptophysin and CD34 were obtained from Ventana (Tucson, AZ). Antibody against GFAP was obtained from Dako (Carpenteria, CA). Antibody against MAP2 was obtained from EMD Millipore (Billerica, MA).

### Tumor Acquisition

Human Rb tumor samples were obtained from enucleated eyes of retinoblastoma patients at the Retinoblastoma Center of Houston's member institutions. For culture, tumor tissue was manually disaggregated and placed in DMEM/F12 50∶50 medium (Mediatech, Manassas, VA) supplemented with either 10% FBS (Gemini Bio-Products, West Sacramento, CA) or B-27 supplement (Life Technologies, Carlsbad, CA). For identification of *RB1* mutations, DNA was obtained from the peripheral blood and the tumor of each patient and sent to Retinoblastoma Solutions (Toronto, ON, Canada) for *RB1* full gene sequencing.

### Cell Culture

To produce tumorspheres, tumor cells were cultured in DMEM/F12 50∶50 medium (Mediatech) supplemented with non-essential amino acids (Mediatech), B-27 supplement (Life Technologies), basic fibroblast growth factor and human epidermal growth factor (STEMCELL Technologies, Vancouver, BC, Canada), as described previously [14]. To produce tumor-derived attached cells, tumor cells were cultured in DMEM/F12 50∶50 medium (Mediatech) supplemented with 10% FBS (Gemini Bio-Products) and 1% penicillin-streptomycin solution (Mediatech). All cultures were maintained in a humidified 37°C incubator with a 95% air, 5% CO_2_ atmosphere.

### RB1 Mutation Analysis

Cultured cells were centrifuged at 500*g for 5 min. The pellet was washed in cold DPBS (Mediatech) and centrifuged again. The pellet was processed to extract total genomic DNA using the DNeasy Blood & Tissue Kit (Qiagen, Valencia, CA) according to the manufacturer's directions. The locus containing the *RB1* mutation was amplified by PCR using the Phusion DNA polymerase kit (New England Biolabs, Ipswich, MA) according to the manufacturer's directions, using primers custom designed to flank each patient's locus. Approximately 100 ng of template DNA per reaction was used, and the reaction was carried out using the following program: 95°C for 5 min.; Repeat 35 times: 95°C for 15 sec., 56°C for 10 sec., and 72°C for 15 sec.; and 72°C for 5 min. The reaction was purified using QIAquick PCR Purification Kit (Qiagen) according to manufacturer directions, and the amplicon DNA was sent to SeqWright, Inc. (Houston, TX) for Sanger sequencing. Chromatogram analysis was performed using FinchTV software (Geospiza, Seattle, WA).

### Immunohistochemistry

To prepare cultured cells for paraffin embedding, cells were pelleted at 1650*g for 10 minutes. After removing the supernatant, the cell pellets were resuspended in 1 mL of fresh frozen plasma. The cells were mixed with 1 mL of Dade Thrombin Reagent (Siemens Healthcare Diagnostics, Tarrytown, NY) and allowed to clot. The clot was placed on lens paper, and excess liquid was removed using gauze. The lens paper containing the clot was folded and place in a tissue cassette. The sample was fixed in 10% formalin for 20 minutes. The fixed cell pellet was embedded in paraffin and sectioned. For staining, sections were deparaffinized and rehydrated, and an antigen retrieval step was carried out by immersion in Target Retrieval Buffer (Dako, Carpinteria, CA) at 95°C for 20 min. Sections were blocked using the Avidin/Biotin Blocking Kit (Vector Laboratories, Burlingame, CA) and Serum-Free Protein Block (Dako) and probed for 30 min. with primary antibody. After washing, biotinylated secondary antibody (Vector Laboratories) was incubated for 30 min. The sections were incubated with VECTASTAIN ABC (Vector Laboratories) for 30 min and stained with DAB chromogen (Dako).

### Statistical Analysis

Synaptophysin-immunolabeled sections were measured for percent area stained with ImageJ software. Statistical significance of synaptophysin stained area was evaluated between tumorsphere and adherent cultures by the Student's t-test. All immunolabeled sections were graded on a four-step ordinal scale based on the percent positive cells, and the median and interquartile ranges were determined. Statistical significance was evaluated in these data sets by the Kruskal-Wallis test coupled with the Dunn post-test. In all cases, significance was assumed at p<0.05.

### Ethics Statement

Human eye tissue and retinoblastoma tumors were obtained under human subject research protocols approved by the Institutional Review Boards at Baylor College of Medicine and The Methodist Hospital Research Institute. Written informed consent was obtained in accordance with the tenets of the Declaration of Helsinki.

## Results

### Only tumorsphere cultures contain RB1 mutations specific to the original tumor

**Figure 1:**
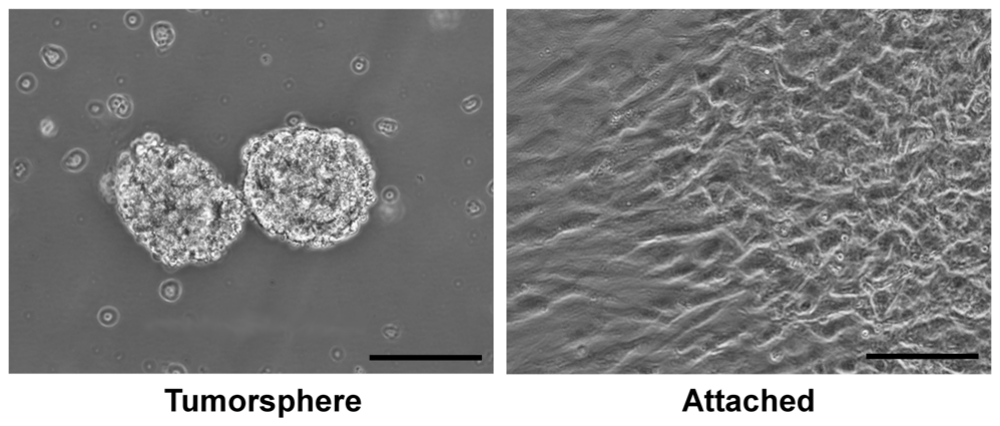
Representative tumorsphere and attached phenotypes of cultures derived from primary retinoblastoma tumor cells. Biopsies from human Rb tumor cells were placed in culture medium. Cultures arose either as three-dimensional spherical aggregates of cells called tumorspheres or as an attached monolayer. Typically, tumorspheres arose when Rb cells were placed in neural stem cell-optimized medium without serum, and adherent cultures arose when Rb cells were placed in classical medium with FBS. However, in rare cases tumorspheres and attached cells both arose from Rb cells placed in classical medium with FBS. Scale bar = 100 µm.

Similar to our previously reported observations [14], [15], when primary tumor samples were placed in neural stem cell medium with defined supplements, tumorspheres arose in culture (Fig. 1). In contrast, tumor samples placed in serum-containing medium produced attached monolayers of cells. Despite the disparate morphologies, visual inspection alone was not sufficient in determining the malignant origin of these primary cultures. To determine whether these primary cultures derive from the malignant cell of origin or from another cell population, sequencing of the *RB1* gene was performed. Patients with identified *de novo* mutations in both alleles of *RB1* in the tumor and at least one wild type *RB1* allele from peripheral blood mononuclear cell DNA (representing the germline genotype) were selected for study. The patients selected presumably developed their tumors through different mechanisms (Table 1). Patient 1 developed Rb by sequentially developing unique mutations in each *RB1* allele and did not appear to contain either mutation in the germ line. Patient 2 developed a large deletion in the *RB1* gene and then, by nondisjunction and reduplication of the 13^th^ chromosome in a tumor-initiating cell, became homozygous for the mutated *RB1* gene. Patient 3 had an *RB1* mutation in the germ line and subsequently developed a second unique mutation in the tumor-initiating cell. Patient 4 developed a mutation in a retinal cell, and subsequent epigenetic silencing by methylation of the remaining normal *RB1* allele resulted in the tumor-initiating cell. Although each of the genetic mechanisms leading to retinoblastoma was unique, the resulting tumors and their ability to either form attached cells or tumorspheres were indistinguishable. Sequences of the *RB1* gene at patient-specific loci show that DNA from tumorsphere cultures contain a mutation in *RB1* identified as unique to the original patient tumor and not found in peripheral blood DNA, while DNA from adherent cultures only contain mutations in *RB1* found in peripheral blood mononuclear cell DNA (Table 1). This observation provides evidence that tumorsphere cultures derive from the malignant cells of the Rb tumor. Importantly, however, genetic analysis shows that attached cells derived from Rb tumors in serum-containing medium conditions typically are not derived from the malignant cells of the tumor.

**Table 1:** RB1 genotypes of primary Rb-derived cultures.

Patient	*RB1* Allele	Tumor	PBMC¶	Tumorsphere	Attached
1	1	c.1072C>T	Normal	c.1072C>T	Normal
	2	IVS12+1G>A	Normal	IVS12+1G>A	Normal
2	1	Δexons 3–16	Normal	Δexons 3–16	Normal
	2	Δexons 3–16	Normal	Δexons 3–16	Normal
3	1	c.1735C>T	Normal	c.1735C>T	Normal
	2	c.1421G>T	c.1421G>T	n.d.§	n.d.
4	1	c.1399C>T	Normal	c.1399C>T	Normal
	2	Methylation	Normal	n.d.	n.d.

¶ peripheral blood mononuclear cell.

§ not done.

### Tumorsphere cultures are primarily pRb-negative and express synaptophysin, while attached cells express pRb and are synaptophysin-negative

**Figure 2:**
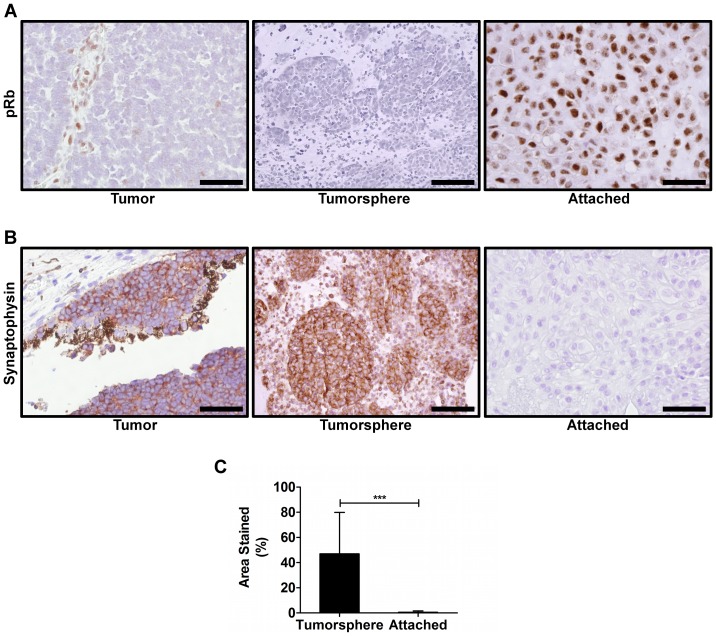
Expression of pRb and synaptophysin in primary retinoblastoma tumor-derived cultures. Stains for pRb and synaptophysin of primary Rb tumor-derived cultures were compared to the expression pattern of the original tumor. **A**. In pRb-negative Rb tumors, the tumor-derived tumorspheres were negative for pRb expression, yet almost all attached cells express pRb (brown). **B**. Tumorspheres diffusely express high levels of synaptophysin similar to the original tumor (brown), while all adherent cultures are negative for synaptophysin expression except one (Table 2). In one adherent culture, <25% of cells were synaptophysin-positive. Scale bar = 50 µm. **C**. Total area of the section staining for synaptophysin was quantified for the tumor-derived cultures (n = 8), and tumorsphere cultures were compared to adherent cultures. Synaptophysin expression was seen in all tumorsphere cultures, and rarely in adherent cultures. Results shown as mean ± SD. *** = p<0.001.

Expression of pRb is lost in many tumors while normal cells retain pRb expression. In those tumors that do retain expression of pRb, the protein in non-functional. Synaptophysin is a synaptic vesicle membrane protein expressed in a punctate pattern in neuronal synapses of the CNS and retina, but is also highly expressed in a diffuse pattern in neuroendocrine malignancies such as Rb [18]. To confirm the malignant origin of tumorsphere cells, tumorspheres and attached cells were paraffin embedded, sectioned and stained for pRb and synaptophysin expression (Fig. 2). In pRb-negative tumors, tumorspheres contained no pRb-positive cells while adherent cultures were highly pRb-positive. In all tumors tested, tumorspheres stained almost 100% positive for synaptophysin expression (Table 2). Importantly, the diffuse cytoplasmic staining pattern is distinct from the normal expression patterns seen in differentiated neurons. Most adherent cultures demonstrated no synaptophysin expression, though one culture showed a subpopulation of synaptophysin-positive cells.

**Table 2:** Expression of tumor and stromal cell markers in Rb-derived cultures.

Patient	Culture	Synapto.¶	Cytoker.§	CD34	GFAP	MAP2
1	Tumorsphere	++++	−	−	−	++++
	Attached	−	++++	−	+	−
2	Tumorsphere	++++	−	−	−	++++
	Attached	+	++	+++	+	+
3	Tumorsphere	++++	−	−	−	++++
	Attached	−	+++	+	−	−
4	Tumorsphere	++++	−	−	−	++++
	Attached	−	++++	−	−	−
5	Tumorsphere	++++	−	−	−	++++
	Attached	−	+	+	−	−

Percent positive cells: − = 0% + = 1–25% ++ = 26–50% +++ = 51–75% ++++ = 76–100%.

¶ synaptophysin.

§ cytokeratin.

### Tumor-derived attached cells express markers of differentiation found in normal retinal cells and stromal cells of the primary tumor

**Figure 3:**
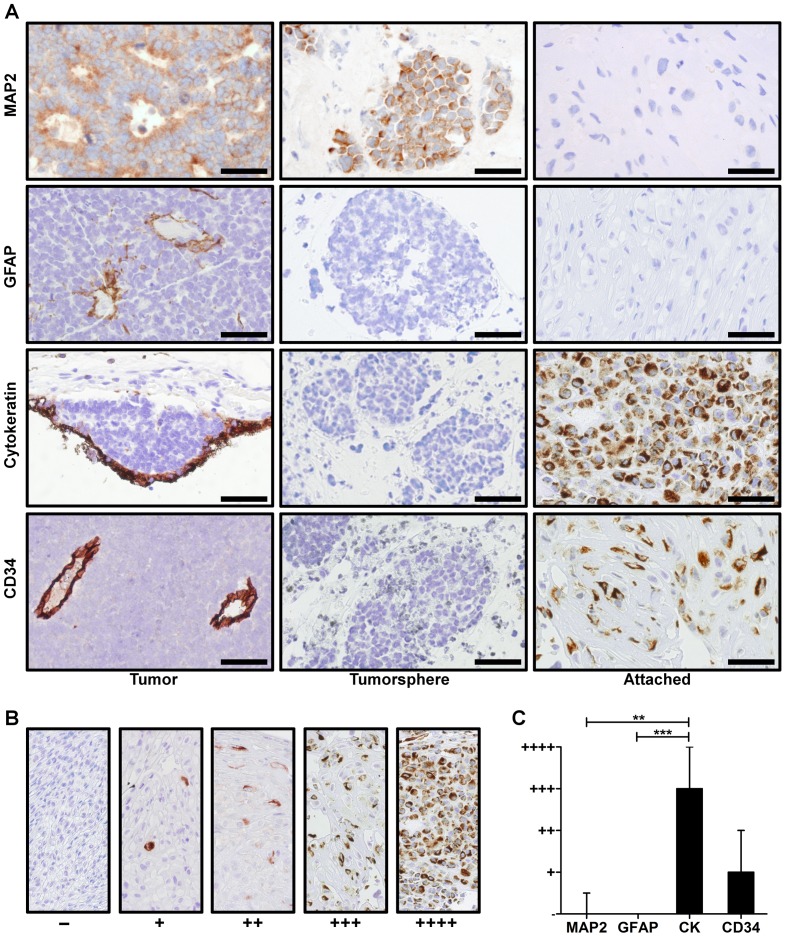
Expression of stromal cell and retinal cell markers in primary retinoblastoma tumor-derived cultures. Expression of MAP2 (n = 5), GFAP (n = 8), cytokeratin (n = 9), and CD34 (n = 5), which identify photoreceptors/ganglion cells, glial cells, retinal pigment epithelial cells and vascular endothelial cells, respectively, was determined in tumor-derived cultures and compared to the original tumor. **A**. Representative staining shows the typical result for each marker (brown). While MAP2 labeled 100% of cells in all tumorsphere cultures, no MAP2-positive cells were seen in all adherent cultures except one (Table 2). In one adherent culture, <25% of cells were MAP2-positive. GFAP, cytokeratin and CD34 were not seen in tumorsphere cultures. Very rare, isolated GFAP-positive cells were seen in only 2 of 5 adherent cultures. Cytokeratin-positive cells were seen in all adherent cultures. A significant CD34-positive subpopulation was seen in 3 of 5 cultures. Scale bar = 50 µm. **B**. Representative sections showing the staining intensity grading (from Table 2) based on approximate number of cells stained. **C**. Quantification of grading of adherent cultures shows higher levels of cytokeratin-positive cells than CD34-positive cells. Results are shown as median ± interquartile range. CK = cytokeratin. ** = p<0.01. *** = p<0.001.

The most likely sources of non-malignant cells in a tumor-derived culture are either stromal cells associated with the tumor or normal retinal cells embedded in the tumor. Immunohistochemistry confirms that Rb tumors express markers of differentiated cell types expected to be found in or near Rb tumors, including epithelial cells of the RPE and vascular endothelial cells. Since non-malignant cells appear to originate from tumor samples, we hypothesized that somatic cells of the retina and tumor stroma are the origin of these cells. To determine the origin of attached cells, tumorspheres and attached cells were paraffin embedded, sectioned and immunolabeled for expression of glial fibrillary acidic protein (GFAP), CD34, and cytokeratin, which are markers of glial cells [19], vascular endothelial cells [20], and epithelial cells [21], respectively (Fig. 3A). Sections were also immunolabeled for microtubule-associated protein 2 (MAP2), which is expressed in photoreceptors and ganglion cells of the retina [22] as well as Rb-derived cell lines [23] (Fig. 3A). Immunolabeled sections were graded based on the percentage of cells that stained positive for each stain (Fig. 3B). All tumorsphere cultures stained 100% for MAP2 expression, while the adherent cultures were, with the exception of one, devoid of MAP2-positive cells (Table 2). Tumorsphere cultures were negative for GFAP, CD34, and cytokeratin, yet all adherent cultures contained significant numbers of cytokeratin-positive cells. Some of the adherent cultures also contained a small population of CD34-positive cells. Only very rare GFAP-positive cells were seen in two of the five adherent cultures. These results suggest that non-malignant cells in the adherent cultures primarily originate from retinal pigment epithelium and vascular endothelium.

## Discussion

Tumors are heterogeneous masses of cells comprised of both malignant cancer cells and non-malignant stromal cells, and it is reasonable to assume that tumor biopsies will typically contain both. This work demonstrates that cultures with disparate growth phenotypes, suspension tumorspheres and adherent monolayers, can arise from primary Rb tumors. The results also demonstrate that, in the establishment of primary cultures derived from Rb tumors, selection of malignant or non-malignant cells may occur in specific culturing conditions. Genetic analysis of *RB1* mutations definitively shows that at least the overwhelming majority of cells in the tumorsphere culture are genetically derived from the malignant cells of the tumor, while almost all cells in the adherent cultures lack the requisite *RB1* mutations of the parent malignant tumor cells. Synaptophysin and MAP2 expression in tumorsphere cultures, as well as staining for retinal cell-specific markers in adherent cultures, provides further evidence that the tumorsphere population derives from malignant cells of the tumor while the attached cells largely derive from non-malignant tumor cells. We previously reported that cone photoreceptor-specific phototransduction enzyme PDE6C was highly expressed in primary Rb tumor yet was significantly reduced in tumor-derived adherent cultures [24]. This work demonstrates that the previously observed reduction was most likely due to non-malignant non-photoreceptor cells from the retina and tumor stroma outnumbering the malignant cells.

The observation that most cells in the adherent cultures are non-malignant would suggest that adherent cultures would show limited growth, since their proliferation would be limited by senescence. We did note that the adherent cultures used in this work persisted to at least 11 passages without evidence of senescence (data not shown). Culture longevity, however, may not be a robust indicator of selection of malignant or non-malignant cells since even cultured primary tumor cells typically exhibit poor long-term persistence in culture [25], [26]. It is important to note as well that in the case of one tumor-derived culture, a minority of attached cells were positive for synaptophysin and MAP2 expression with localization inconsistent with ganglion cells or photoreceptors, implying that in rare instances malignant cells may persist in attached culture conditions. The persistence of a small malignant subpopulation within some adherent cultures suggests that those cultures may exhibit tumorigenicity when introduced into animals. Additionally, retinoblastoma-derived cell lines WERI-Rb1 and Y79, both of which have been validated regarding pRb expression and *RB1* mutation [27], [28] and grow as cellular clusters in suspension, were isolated and are cultured in serum-containing medium used to culture non-malignant primary cells in this study. As with other primary tumor cultures such as breast cancer [26], the successful long-term culturing of Rb-derived Y79 cell line was a rare success among many failed attempts [25], and our observation of a malignant sub-population in one attached primary culture is consistent with the tendency in the history of cell culture of the successful isolation of a persistent cell line in standard culture medium to be infrequent. Furthermore, we observe that tumorspheres arise in attached culture conditions less frequently than in stem cell-optimized medium, suggesting that underlying genetic or epigenetic variations in a particular patient or malignancy may allow for persistence of these malignant cultures in serum-containing medium.

In the study presented here we have identified culturing conditions that promote the growth of distinct tumor subpopulations. In all cultures evaluated for this study, suspension tumorsphere cultures are comprised of malignant cells, providing a definitive method for reliably culturing malignant tumor cells *in vitro*. In contrast, attached cell cultures are predominantly comprised primarily of non-malignant stromal cells. Genetically determining the origin of cells in culture is not feasible in most situations. Retinoblastoma, by virtue of its etiology requiring mutations of both *RB1* genes, facilitates easy tracking of the origins of cells grown outside of the original tumor by sequencing the *RB1* gene and comparing the results to clinical genetic sequencing of both the original tumor and the germline. Only in very rare instances, such as in benign retinocytomas [29], do non-malignant cells lose all functioning pRb. It is important to note, however, that while this study highlights the tendency of the selection of these cell populations under certain conditions, it does not preclude the occurrence of malignant cell cultures arising in serum-containing medium conditions. It is impossible to definitively conclude that similar results as presented here will occur with all tumors in all conditions. Nevertheless, the observations presented here remain significant even if selection of non-malignant cells in culture only occurs occasionally. Researchers may not be able to distinguish between a successful isolation of malignant cells without some method of validation similar to the ones presented in this work, and even the sporadic utilization of non-malignant cells in experiments would yield inconsistent or flawed results. Our observations demonstrate that care must be exercised in utilizing primary tumor-derived cultures for *in vitro* experimentation to ensure that the results are applicable to the malignancy being studied. As whole-genome sequencing becomes increasingly cost-effective, validation of tumor-derived cultures will become more practical for malignancies other than Rb.
